# NATURAL DISASTERS: Arsenic Spike from Ike

**DOI:** 10.1289/ehp.117-a294a

**Published:** 2009-07

**Authors:** Naomi Lubick

**Affiliations:** **Naomi Lubick** is a freelance science writer based in Zürich, Switzerland, and Folsom, California. She has written for *Environmental Science & Technology, Nature, and Earth*

A study of the sediment left behind by flooding from Hurricane Ike in September 2008 shows that although levels of most of the toxic compounds assayed are low, some concerns remain about elevated arsenic levels. The larger impact, however, may come from the cooperation between scientists and community members, whose combined effort has yielded valuable insights as coastal communities gear up for the 2009 hurricane season.

To determine exactly what Hurricane Ike delivered, researchers from the University of Texas Medical Branch (UTMB) in Galveston joined local volunteers from community organizations to collect sediment samples. The samples, collected from hard surfaces to distinguish flood-borne sediment from pre-existing soil, were analyzed at a nearby laboratory. The results showed mostly “good news,” the team reported in the February 2009 white paper “Analysis for Toxic Materials in Sediments from Hurricane Ike in Galveston, TX.” The sediment did not contain sufficient levels of most pollutants measured—such as polychlorinated biphenyls, dioxins, and petroleum products—to require remediation.

However, several sites had elevated levels of some metals, including arsenic at amounts up to 48 times the U.S. Environmental Protection Agency’s residential screening level, although below the level that legally requires cleanup. Moreover, the highest arsenic levels were found in a particularly poor neighborhood. “Without adequate information and self-protection,” the team wrote, “this community will likely suffer a heavier exposure burden, and may be most vulnerable to the effects of that burden.”

The report is not comprehensive, the team acknowledges; the nine samples collected cannot show the complete picture, and the analytical tests were basic. However, with the resources at hand (and a $7,500 budget), the minimal data set is enough to support the team’s basic conclusions: floodwaters tend to stir up what’s already there, while sometimes adding more contaminants from elsewhere, depending on where the floodwaters originated.

Some likely sources of the arsenic in Galveston include now-defunct industrial sites, says project co-investigator Jonathan Ward. An old tin smelter across Galveston Bay, for example, once dumped its waste into a channel that directly flowed into the estuary, Ward says. Co-investigator Sharon Petronella says the UTMB team wants to map out such “legacy” contaminants in a geographic information system to serve as a “predecessor for a risk assessment working backwards.”

Howard Mielke, a research professor at Tulane University who has documented lead-laden soils of New Orleans and other cities, notes that such a historical record would have been useful in assessing Hurricane Ike flood deposits. With 22% of the Galveston samples containing lead levels over 200 ppm, Mielke says the post-Ike lead content seems relatively high. He adds, “Because of their extraordinary vulnerability, no child should be playing in soil with a lead content above 100 ppm.”

The sediment monitoring project is part of UTMB’s LEAST Lead Initiative, which seeks to reduce lead poisoning in Galveston in cooperation with county and state agencies and with St. Vincent’s House, a nonprofit social service agency. The impetus for the monitoring project came from St. Vincent’s executive director Michael Jackson, who rallied support at a meeting in the gutted bottom story of the agency a few weeks after Ike struck. Petronella says, “If you don’t have a community voice at the table, you are not going to understand what the community needs.” Community involvement such as having residents tell their neighbors how to protect babies from hand-to-mouth exposure had been important to LEAST Lead’s efforts prior to Hurricane Ike and proved equally effective with regard to exposure to flood debris and sediments.

Researchers outside the project agree that the collaborative nature of the work is a positive highlight of the report. Research geochemist Geoff Plumlee of the U.S. Geological Survey, who assessed New Orleans flood deposits after Hurricane Katrina in 2005, says the report “actually got me thinking about things we could do at the local grass-roots level, because that’s clearly where the impacts are.”

